# Autosomal short tandem repeat genetic variation of the Basques in Spain

**DOI:** 10.3325/cmj.2011.52.372

**Published:** 2011-06

**Authors:** Kristin L. Young, Guangyun Sun, Ranjan Deka, Michael H. Crawford

**Affiliations:** 1 Department of Family Medicine, Research Division, University of Kansas Medical Center, Kansas City, Kan, USA; 2 Department of Environmental Health, University of Cincinnati Medical Center, Cincinnati, Ohio, USA; 3 Department of Anthropology, University of Kansas, Lawrence, Kan, USA

## Abstract

**Aim:**

To examine population genetic structure and hypotheses of the origin of the modern Basque population in Spain using autosomal short tandem repeat (STR) data from individuals living in 27 mountain villages in the provinces of Alava, Vizcaya, Guipuzcoa, and Navarre, by comparing Basque autosomal STR variation with that of neighboring populations in Europe, as well as proposed ancestral populations in North Africa and the Caucasus.

**Methods:**

Allele frequencies for 9 autosomal STR loci (D3S1358, D5S818, D7S820, D8S1179, D13S317, D18S51, D21S11, FGA, and vWA) and several population genetic parameters were determined for the 4 provinces in the Basque region of Spain (n = 377). Heterozygosity within the Basque population was measured using a locus-by-locus analysis of molecular variance. Relationships between the Basques and other populations were examined using a multidimensional scaling (MDS) plot of Shriver’s D_SW_ distance matrix.

**Results:**

Heterozygosity levels in the Basque provinces were on the low end of the European distribution (0.805-0.812). The MDS plot of genetic distances revealed that the Basques differed from both the Caucasian and North African populations with respect to autosomal STR variation.

**Conclusions:**

Autosomal STR analysis does not support the hypotheses of a recent common ancestor between the Basques and populations either from the Caucasus or North Africa.

The question of Basque origins has interested scholars since the 1800s, when Aranzadi suggested, based on cranial morphology, that the Basques were an ancient relict population (1). The Basque language, *Euskara*, is most widely accepted as an isolate, unrelated to any other extant language in Europe (2). Many hypotheses of a relationship between Basques and other populations have been put forward based on linguistic analyses. These proposed linguistic connections include the ancient languages of Iberian, Minoan, Etruscan, Pictish, Sumerian, and Aquitanian, as well as extant Uralic (such as Finnish), Caucasian (such as Georgian), African (especially Berber), and Native American languages, and even Japanese (3-10). It has been suggested that populations in the Basque region and the Caucasus, which speak non-Indo-European agglutinative languages, could be remnants of a Mesolithic European population and have been less affected than the rest of the continent by the Neolithic Revolution for the same reason – both inhabit mountainous regions that were less hospitable to agricultural pursuits (6). Alternatively, the Vasco-Iberian hypothesis holds that languages related to Basque were spoken throughout the Iberian Peninsula prior to Roman conquest. A genetic relationship between *Euskara* and Iberian was favored in the late 1700s, with Basque considered the last remnant of this larger language family, but discoveries of Iberian inscriptions which were not translatable using *Euskara* weakened this hypothesis on linguistic grounds. Because Iberians were believed to have migrated from North Africa, and a connection between Iberian and Basque had been proposed, genetic similarities between Basques and North Africans have also been sought (11). There are also linguists who conclude that Basque is an autochthonous language, which developed in situ in the Iberian Peninsula, and once had a wider range, but has also had contact with other languages in historical times (2).

Studies of human blood types in the mid-20th century bore out the distinctiveness of the Basques, distinguishing them from other European populations by a low frequency of ABO*B (1.1%) and a high frequency of RH*cde (between 30.5%-35.6%) (12-15). Since then, the Basque population has been characterized from a genetic perspective using blood group antigens (12-20), erythrocytic enzymes (21-23), plasma proteins (24), HLA antigens and haplotypes (25-30), Y-chromosome markers (31-36), mitochondrial haplogroups and sequences (37-44), whole genome single-nucleotide polymorphism (SNP) analyses (45-47), and autosomal microsatellites (48-58). Microsatellites are sequences of 2-6 bases tandemly repeated 10-30 times, which are found scattered throughout the genome. These short tandem repeats (STR) are considered selectively neutral, and therefore appropriate for population genetic studies. Thirteen of these STR loci comprise the Combined DNA Index System (CODIS) used for forensic purposes, and have been widely implemented in anthropological genetics because forensic databases provide a wealth of comparative data.

The present study characterizes autosomal STR genotypes from the Basque region of Spain to examine population substructure and genetic relationships with other groups, including testing of the proposed genetic affinities between the Basques and populations in the Caucasus and North Africa. We predict that if the Basques share a common ancestor (or have experienced more recent migration and resultant gene flow) with either populations in the Caucasus or North Africa, allele frequencies of autosomal STR loci will be similar among the Basques and these proposed related populations and genetic distances between these populations will be low. Alternatively, if the Basques are an autochthonous European population (with no recent gene flow from groups in North Africa or the Caucasus), autosomal STR frequencies will be within the range of other populations on the European continent, and the Basques will be more genetically similar to other European groups. Previous studies presenting STR data from the Basque population either used small samples that were often collected in urban areas of a single province (when collection location was reported) to study relationships between the Basques and other populations (49,54,58-60). When larger samples were collected (48,50-52,57), the studies most often presented allele frequencies and population genetic parameters for only a few loci (Table 1). This study represents one of the most comprehensive samples of Basques yet analyzed for autosomal STR variation, with 377 individuals in 27 mountain villages from throughout the Basque region of Spain.

**Table 1:** Previous autosomal short tandem repeat studies in the Basque population.

Population	Location	N	Loci	Analyses	Reference
Rural autochthonous Basque females	Rural Basque region	57	1	allele frequencies, population genetic parameters, genetic distances, neighbor-joining (NJ) tree	(49)
Autochthonous Basques	Basque Country	326	6	allele frequencies	(52)
Basque autochthonous residents	Guipuzcoa	50	13	allele frequencies, population genetic parameters	(54)
Unrelated autochthonous Basques	Basque Country	206	5	allele frequencies, population genetic parameters	(51)
Basque Country autochthonous individuals	Basque Country	202-208	7	allele frequencies, population genetic parameters	(50)
Unrelated “native Basques”	Alava	101	13	allele frequencies, population genetic parameters, forensic parameters	(57)
Unrelated Basque country residents	Basque Country	100	3	allele frequencies, population genetic parameters, population comparison	(48)
Unrelated autochthonous Basque students	Navarre	107	3	allele frequencies, population genetic parameters, population comparison	(59)
Unrelated autochthonous Basques	Basque Country	200	3	allele frequencies, population genetic parameters, forensic parameters, population comparison	(60)
Unrelated autochthonous Basques	Vizcaya	73	13	allele frequencies, population genetic parameters, forensic parameters, NJ tree	(56)
Unrelated autochthonous Basques	Vizcaya	68	9	allele frequencies, population genetic parameters, NJ tree, multidimensional scaling plots	(58)

## Materials and methods

### Population

**Figure 1:**
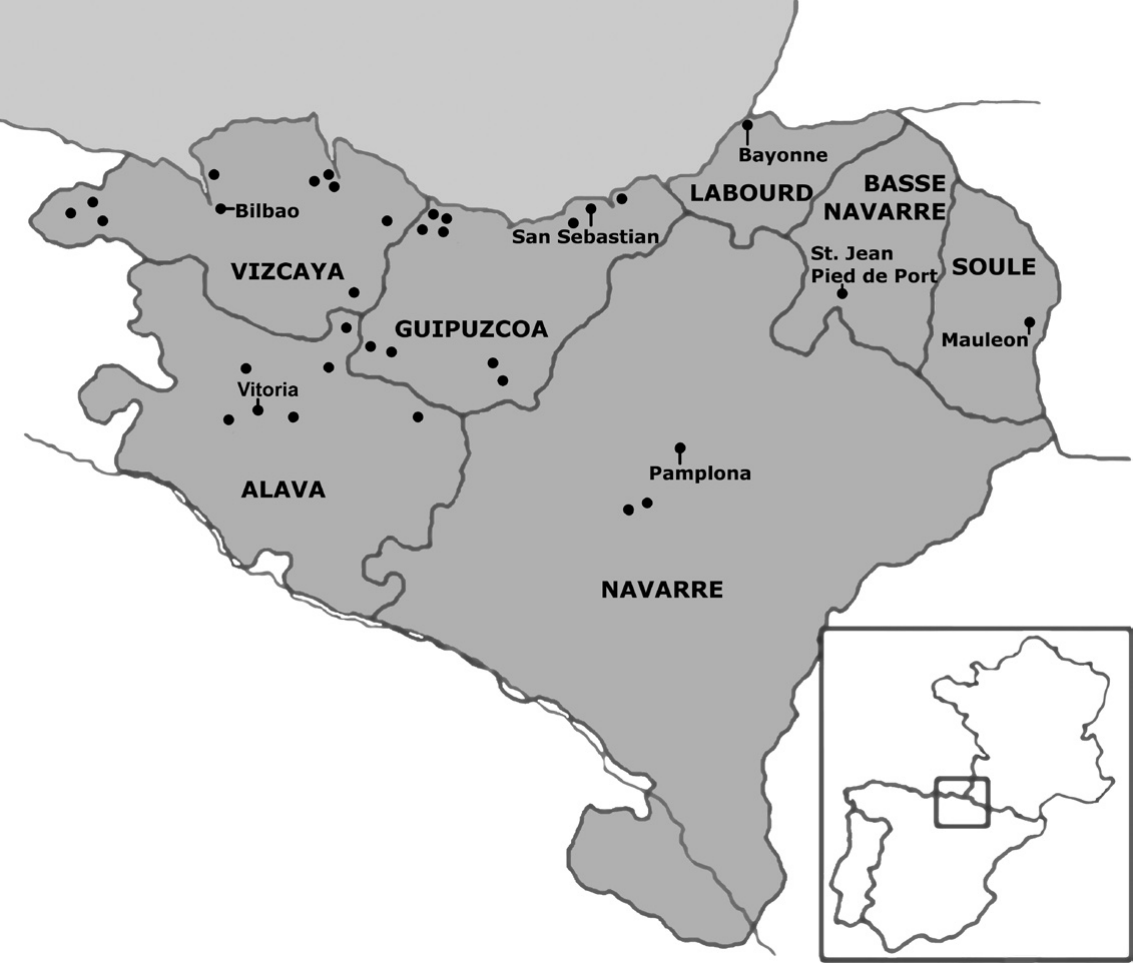
Map of the Basque provinces in France and Spain. Sampling locations – black circles, provincial capitals – ball-and-stick.

To test the hypotheses of genetic similarity between Basques and other populations, buccal DNA samples were collected from 652 autochthonous (those who claimed 4 Basque grandparents) participants of both sexes, in mountain villages throughout the Basque region of northern Spain. Six villages were sampled in Alava (n = 143), 17 villages in Vizcaya (n = 237), 10 villages in Guipuzcoa (n= 220), and 2 villages in Navarre (n = 52). A sub-sample of individuals from 27 villages (n = 478) was screened for autosomal STR genotypes (Table 1) (Figure 1). The samples were collected during summer field seasons between 2000 and 2002. Lab analysis was performed in 2003-2004, and statistical analysis was conducted between 2005-2007 (with some additional analyses done as part of KY’s dissertation in 2009 and revised analyses for publication in 2011). This study was approved by the University of Kansas Human Subjects Committee (HSCL #11955), and participants provided written informed consent.

### DNA analysis

DNA extraction was performed using a standard phenol:chloroform protocol. A portion of each sample was reserved for autosomal STR analysis using the Applied Biosystems Profiler Plus Kit (Foster City, CA; USA). The samples were characterized for 9 STR loci, including D3S1358, D5S818, D7S820, D8S1179, D13S317, D18S51, D21S11, FGA, and vWA, plus the sex-determining amelogenin locus, using a multiplex PCR procedure according to manufacturer’s instructions (61). Amplified products were detected using an ABI 377 DNA sequencer. DNA fragments were sized and genotyped with GeneScan 3.1 and Genotyper 2.5 software (Applied Biosystems).

### Comparative population data

To test hypotheses of population origins, autosomal STR data from our sample were compared to geographically and/or proposed related populations, including others from the Iberian Peninsula (Andalusia, Cantabria, Catalonia, Galicia, Murcia, Portugal, and Valencia) (54,62-67); Europe (Austria, Belgium, Bosnia, Germany, Greece, Hungary, Tuscany, Poland, Russia, Scotland, Serbia and Montenegro, Slovenia, and Switzerland) (68-81); North Africa (Egypt, Morocco) (82,83); the Middle East (Turkey) (84); and the Caucasus (Georgia) (85).

### Statistical analysis

Allele frequencies were estimated using the gene counting method. Expected heterozygosity under Hardy-Weinberg equilibrium was estimated by the method of Guo and Thompson (86), and locus-by-locus analysis of molecular variance (AMOVA) was performed to examine genetic substructure among the Basques, using Arlequin 3.11 software (87). Genetic differentiation between the Basques and 27 comparative populations, including 1 Caucasian and 2 North African groups, was measured using heterozygosity and gene differentiation (G_ST_). G_ST_ and gene diversity values for the Basques and comparative populations were calculated using DISPAN software (88). Genetic distances between populations were calculated using Shriver’s distance (D_SW_), an adaptation of Nei’s standard distance (D) weighted by the difference in number of repeats between alleles to account for the stepwise mutation pattern of tandem repeat loci. Genetic relationships between groups were examined using a multidimensional scaling (MDS) plot of the genetic distance matrix in NTSYS 2.1 software (89). MDS stress values were evaluated using the criteria of Sturrock and Rocha (90).

## Results

Examination of the autosomal STR data revealed that allelic dropout occurred in 21% of the sample (101 of 478), so that not all loci were amplified for every individual. Samples that did not amplify for all loci were removed from the analysis. Allele frequencies for the autosomal STR loci in each of the Basque provinces after correction for allelic dropout are available in the web extra material.(web extra material 1) 

Observed heterozygosity values among the Basques ranged from 0.60526 (D5S818) to 0.92105 (vWA), with both extremes found in Navarre, likely as a result of small sample size (N = 38) (Table 2). In Alava and Vizcaya, only 2 loci had significantly lower heterozygosity values than expected. When the Bonferroni correction for multiple tests was applied, only the D8S1179 locus demonstrated an excess of homozygotes in all provinces, and bolded *P* values indicate those loci with lower than expected heterzogosity after correction of the data for allelic dropout (Table 2). This suggests that for the other STR loci examined, the expectations of Hardy-Weinberg equilibrium were met (Table 2).

**Table 2:** Exact test of Hardy-Weinberg Equilibrium for 9 autosomal loci in 4 Basque Provinces. Significant *P*-values are in bold*

Locus	Alava	Vizcaya	Guipuzcoa	Navarre
D3S1358	n = 96	n = 89	n = 154	n = 38
*H_O_*	0.77320	0.76404	0.76623	0.68421
*H_E_*	0.80615	0.77433	0.77829	0.80000
*P*	**0.02230**	0.28137	0.34904	0.57454
FGA				
*H_O_*	0.87500	0.85393	0.81818	0.81579
*H_E_*	0.86044	0.88574	0.87578	0.87930
*P*	0.90451	**0.01880**	0.12451	0.14558
D5S818				
*H_O_*	0.65625	0.79775	0.72727	0.60526
*H_E_*	0.70610	0.72780	0.74159	0.73439
*P*	0.14642	0.82662	0.65212	0.42251
D7S820				
*H_O_*	0.71875	0.77528	0.78571	0.73684
*H_E_*	0.80928	0.81553	0.80132	0.82596
*P*	0.07874	0.32608	0.29700	0.70250
D8S1179				
*H_O_*	0.77083	0.76404	0.75325	0.86842
*H_E_*	0.80988	0.80956	0.81006	0.83474
*P*	**<0.00001**	**<0.00001**	**0.00082**	**0.04749**
vWA				
*H_O_*	0.85417	0.82022	0.79870	0.92105
*H_E_*	0.81086	0.81946	0.79864	0.82561
*P*	0.05728	0.88346	0.07616	0.43085
D13S317				
*H_O_*	0.76042	0.76404	0.75325	0.71053
*H_E_*	0.76167	0.79794	0.78282	0.76596
*P*	0.27417	0.11321	0.80221	0.56594
D18S51				
*H_O_*	0.80208	0.79775	0.81818	0.81579
*H_E_*	0.86938	0.88282	0.87571	0.87053
*P*	0.12041	0.09120	0.24056	0.86468
D21SS11				
*H_O_*	0.89583	0.83146	0.80519	0.86842
*H_E_*	0.84004	0.84067	0.84073	0.82246
*P*	0.97004	0.05701	0.50981	0.53438

*Abbreviations: H_O_ – observed heterozygosity; H_E_– expected heterozygosity.

The results of the AMOVA (Table 3) suggested no obvious genetic structuring between provinces, as indicated by the among-groups covariance component (V_a_ = -0.095). The lack of structure among provinces was confirmed by the global estimate of the fixation index among groups (F_CT_ = -0.0036, *P* = 0.892). A small amount of subdivision was found between villages within provinces (1.309% total variation, F_SC_ = 0.0131, *P* = 0.001). Examination of the locus-by-locus results revealed that 3 loci made significant contributions to the differences between villages: D7S820 (F_SC_ = 0.0332, *P* = 0.023), vWA (F_SC_ = 0.0185, *P* = 0.045), and D18S51 (F_SC_ = 0.0319, *P* = 0.021). The majority of variation, however, was found between individuals within villages (99% total variation).

**Table 3:** Locus-by-locus analysis of molecular variance of 9 autosomal short tandem repeat loci. Negative values result from the manner in which the covariance components are estimated, from the mean squares and lower level variances rather than as sums of squares (91)*

	Among groups			Among populations			Within populations		
Locus	percent variance	F_CT_	*P*	percent variance	F_SC_	*P*	percent variance	F_ST_	*P*
D3S1358	-0.026	-0.0003	0.353	1.257	0.0126	0.142	98.768	0.0123	0.153
FGA	-0.161	-0.0016	0.677	-0.591	-0.0059	0.814	100.752	-0.0075	0.849
D5S818	0.384	0.0038	0.182	-0.292	-0.0029	0.646	99.908	0.0009	0.544
D7S820	-1.036	-0.0104	0.939	3.350	0.0332	0.023	97.686	0.0231	0.032
D8S1179	-0.564	-0.0056	0.904	0.573	0.0057	0.203	99.990	0.0001	0.322
vWA	0.011	0.0001	0.371	1.851	0.0185	0.045	98.138	0.0186	0.045
D13S317	-0.318	-0.0032	0.641	0.110	0.0011	0.497	100.208	-0.0021	0.614
D18S51	-0.546	-0.0055	0.504	3.204	0.0319	0.021	97.342	0.0266	0.010
D21S11	-0.308	-0.0031	0.602	0.767	0.0076	0.316	99.541	0.0046	0.321
Global estimates	-0.355	-0.0036	0.878	1.309	0.0131	0.011	99.045	0.0096	0.019
Covariance estimates	V_a_ = -0.095			V_b_ = 0.351			V_c_ = 26.517		


*Abbreviations: F_CT_ – fixation index among groups; F_SC_ – fixation index among populations within groups; F_ST_ – fixation index within populations.

Average heterozygosity values by population ranged from 0.803 in Morocco to a high of 0.820 in Scotland (Table 4). Among the Basques, heterozygosity was lowest in Alava (0.805) and highest in Vizcaya (0.812). This was within the range of heterozygosity values seen in other modern Iberian populations (0.804-0.815). Total gene diversity between subpopulations (H_T_) was high, ranging from 0.724 for D5S818 to 0.878 for D18S51. However, most of this diversity is explained by variation between individuals within subpopulations (H_S_). The percentage of gene differentiation between subpopulations relative to the total gene differentiation (Gst) ranged from a high of 0.009 for D13S317 and D21S11 to a low of 0.006 for D3S1358, FGA, D7S820, vWA, and D18S51.

**Table 4:** Gene diversity between populations based on autosomal short tandem repeat data

Population	D3S1358	FGA	D5S818	D7S820	D8S1179	vWA	D13S317	D18S51	D21S11	Average
Alava*	0.804	0.860	0.705	0.808	0.790	0.807	0.758	0.869	0.840	0.805
Vizcaya*	0.772	0.886	0.728	0.816	0.781	0.817	0.792	0.876	0.840	0.812
Guipuzcoa*	0.778	0.875	0.741	0.801	0.801	0.799	0.773	0.876	0.840	0.809
Navarre*	0.798	0.879	0.705	0.826	0.815	0.826	0.754	0.870	0.819	0.810
Andalusia	0.803	0.868	0.708	0.797	0.824	0.805	0.795	0.879	0.856	0.815
Cantabria	0.796	0.871	0.715	0.796	0.826	0.803	0.779	0.882	0.846	0.813
Catalonia	0.785	0.860	0.714	0.815	0.781	0.825	0.769	0.879	0.809	0.804
Galicia	0.786	0.855	0.712	0.796	0.817	0.822	0.795	0.880	0.830	0.810
Murcia	0.815	0.860	0.718	0.787	0.807	0.820	0.758	0.866	0.826	0.806
Valencia	0.800	0.872	0.702	0.803	0.826	0.807	0.781	0.875	0.839	0.812
Austria	0.806	0.864	0.709	0.808	0.814	0.806	0.801	0.872	0.854	0.815
Belgium	0.801	0.854	0.707	0.811	0.806	0.808	0.793	0.880	0.831	0.810
Bosnia	0.794	0.850	0.713	0.803	0.815	0.807	0.745	0.879	0.867	0.808
Germany	0.781	0.870	0.709	0.814	0.790	0.818	0.774	0.885	0.841	0.809
Greece	0.788	0.855	0.733	0.794	0.814	0.822	0.775	0.881	0.846	0.812
Hungary	0.794	0.864	0.728	0.798	0.809	0.805	0.787	0.886	0.855	0.814
Tuscany	0.788	0.865	0.722	0.796	0.832	0.793	0.745	0.868	0.852	0.807
Poland	0.802	0.863	0.717	0.812	0.797	0.804	0.759	0.873	0.866	0.810
Portugal	0.786	0.862	0.710	0.811	0.816	0.810	0.785	0.876	0.848	0.811
Russia	0.783	0.860	0.734	0.811	0.799	0.803	0.781	0.878	0.845	0.810
Scotland	0.799	0.856	0.727	0.804	0.834	0.811	0.828	0.864	0.855	0.820
Slovenia	0.797	0.876	0.718	0.810	0.780	0.809	0.785	0.879	0.855	0.812
Switzerland	0.791	0.869	0.725	0.821	0.830	0.809	0.773	0.877	0.842	0.815
Egypt	0.772	0.873	0.763	0.784	0.819	0.806	0.792	0.858	0.825	0.810
Morocco	0.779	0.851	0.727	0.772	0.824	0.822	0.748	0.878	0.831	0.803
Turkey	0.780	0.864	0.751	0.813	0.822	0.802	0.779	0.872	0.842	0.814
Georgia	0.775	0.871	0.749	0.810	0.806	0.765	0.746	0.874	0.855	0.806
H_S_^†^	0.788	0.862	0.720	0.801	0.808	0.807	0.773	0.872	0.839	0.807
H_T_^‡^	0.792	0.866	0.724	0.806	0.814	0.812	0.780	0.878	0.847	0.813
G_ST_^§^	0.006	0.006	0.007	0.006	0.008	0.006	0.009	0.006	0.009	0.007

*Present study.

†Gene diversity within subpopulations.

‡Gene diversity between subpopulations.

§Coefficient of gene differentiation (92).

**Figure 2:**
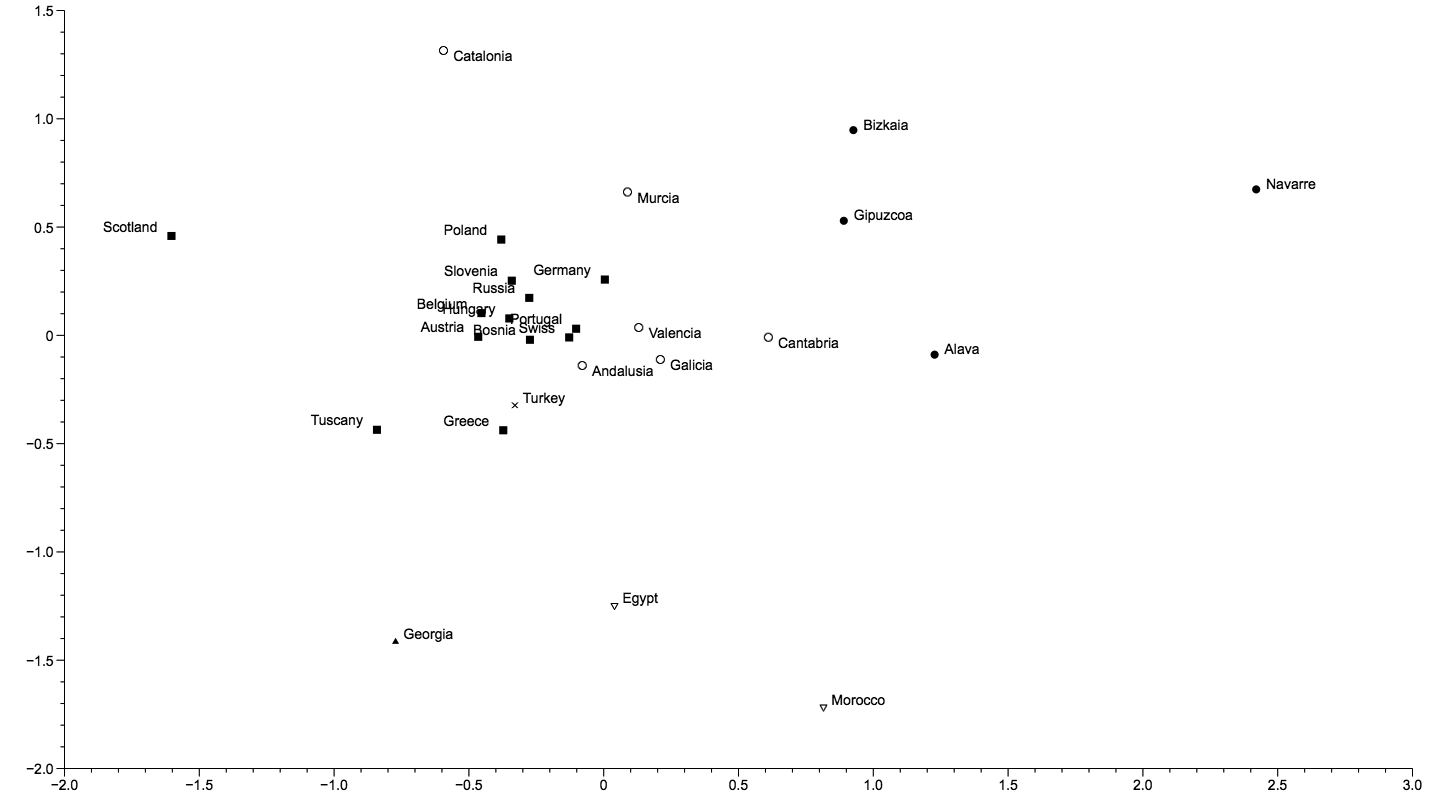
Multidimensional Scaling plot of genetic distance between 27 populations (Basques – closed circles, Iberia – open circles, Europe – closed squares, Middle East – cross, Caucasus – closed triangles, North Africa – open triangles). The first two axes account for 26.02% of the total genetic variation present in the sample. The stress value of 0.169, well below the threshold of 0.317 for 27 populations in 2 dimensions (90), demonstrates that the plot is an accurate representation of the genetic distance matrix. A Mantel test of matrix correlation between the original distance matrix and the MDS matrix also demonstrated that the MDS plot was an accurate represent of the genetic distances between populations (correlation coefficient: *r* = 0.93498, *t* test: *t* = 5.7717, *P* = 1.0). The Basque groups cluster together on the right side of the plot, near their neighbors in Cantabria. The North African and Georgian populations are found near the bottom center of plot, differentiated from the other European groups.

Visual representation of genetic distances between populations using multidimensional scaling (Figure 2) showed that the Basque groups clustered together on the right side of the plot, near their neighbors in Cantabria. The North African and Georgian populations were found near the bottom center of plot, differentiated from the other European groups. A stress value of 0.169, well below the threshold of 0.317 for 27 populations in two dimensions, demonstrates that the plot is an accurate representation of the genetic distance matrix. A Mantel test of matrix correlation between the original distance matrix and the MDS matrix also demonstrated that the MDS plot was an accurate represent of the genetic distances between populations (correlation coefficient: *r* = 0.93498, *t* test: *t* = 5.7717, *P* = 1.0).

## Discussion

Our study of the autosomal STR variation did not support the hypotheses of a recent common ancestor between the Basques and populations either from the Caucasus or North Africa. Allelic dropout was noted for several samples in the present analysis, raising the possibility of a technical or genomic error in the typing of samples. At low sample DNA concentrations, the Profiler Kit is known to preferentially amplify short alleles and homozygotes (93). Because the samples collected in this study were from buccal swabs, and only a portion of each sample was used for STR analysis, DNA concentrations were much lower than if the samples had been from whole blood. The excess of homozygotes at D8S1179, even after correction for allelic dropout, was of particular concern. Concordance studies of autosomal STR typing across different multiplex kits have reported issues with the D8S1179 locus in certain populations, principally with alleles 15-18 using the Profiler Plus Kit (94). Reports of D8S1179 from previous studies among Basques give frequency ranges for alleles 15-17 between: 15 (0.110-0.210), 16 (0.010-0.029), and 17 (0.005-0.007) (54,56-58). D8S1179*18 has not been previously reported in Basques. Frequencies for D8S1179 alleles 15-17 in the present study fall within the ranges previously reported for this population (54,56-58), and we also found no individuals with allele 18. In addition, the locus-by-locus AMOVA demonstrated that the Basque provinces were homogeneous with respect to autosomal STR variation, and the single locus found to not be in HWE (D8S1179) did not significantly contribute to differences between provinces. Therefore, we do no believe that the failure to meet HWE in this case represents a technical error, and we included the D8S1179 allele in the interpopulation analyses.

The present analysis of autosomal STR variation does not support either the Caucasian or Vasco-Iberian hypothesis of Basque origins. Caucasian languages themselves are not a cohesive group, and while some linguists see similarities between Basque and some aspects of the northern or southern Caucasian languages, these similarities have been attributed either to poor interpretation, a shared Euro-African substratum, or similarities in the evolution of language itself (11). Examination of the literature on the Basque-Caucasian hypothesis demonstrates little support from the genetic evidence (6,23,95-98). Cluster analysis of classical genetic markers showed that subpopulations sampled in Vizcaya were more genetically similar to each other than to other European populations or Caucasian groups outside Europe, such as those in Asia Minor and the Middle East (99). Comparison of Basque and populations from the Caucasus using 10 blood group and serum protein loci revealed that both non-Indo-European groups were more genetically similar to their neighbors than to each other (6). Analysis of HLA data showed that the Svani (a Kartvelian-speaking population) and the Basques were found to share only a single five-locus extended haplotype, A*01-B*8-DRB1*03-DQA1*0501-DQB1*0201 (95). This is the most frequent HLA haplotype found in Europeans (100,101), and is present in the Svani at a frequency of 1.25% and among Basques at 2%, leading the authors to conclude that the HLA system does not support the hypothesis of a relationship between these groups.

Recent studies of molecular markers also found little similarity between Basques and populations living in the Caucasus region. Analysis of Y-SNP haplogroups found that F_ST_ values between Basques and Caucasus-dwelling groups were much greater than between Basques and surrounding Indo-European populations (96). While comparison of mtDNA sequences did reveal greater affinity between European groups and Caucasians than between West Asians and Caucasians (97), the addition of populations from Iran resulted in a genetic picture in which the Caucasus groups fell between populations from Europe and Asia Minor with respect to mtDNA sequence variation (98). As with Y-SNPs, genetic distances based on mtDNA sequences were greater between Basques and Caucasians than between Basques and Indo-Europeans, lending credence to the hypothesis of no genetic relationship between Basques and Caucasian populations. The results of the present study agree with those previously published using other genetic markers, as genetic distances based on autosomal data place the Basque groups in a different quadrant of the MDS plot than the population from Georgia.

The Vasco-Iberian hypothesis is based partly on craniometrics, the anthropometry of head shape (102). Broca suggested, based on a sample of 60 skulls from Guipuzcoa, that the Basques were similar to populations in North Africa (103,104). However, a reanalysis of Broca’s sample supplemented by the addition of 19 skulls noted no greater similarity between Basques and North African groups than any other European populations with regards to head shape (105). A more recent multivariate analysis of 20 craniometric variables in 13 Iberian populations demonstrates the unique position of Basques in the Iberian Peninsula (106). Regardless of sex, Basques were distinct in every analysis performed. The differences between Basques and other Iberian populations could not be accounted for solely by geographic distance and were instead attributed to greater age of the Basque population relative to the others.

The majority of genetic studies supporting a relationship between Basques and North African populations have been based on HLA data (8,107-111). Other genetic systems do not support a relationship between Basques and North African groups (112-114), and additional HLA analyses also found no evidence of a relationship between the two populations (29,115-119). Preliminary investigation of autosomal STRs in Vizcaya Province indicated similarity with the Basque province of Guipuzcoa, and distinction from North African groups in the Maghreb (58). The present study demonstrates the lack of relationship between Basques and populations of North Africa, as the Basque populations do not cluster near either North African population included in the MDS plot (Morocco and Egypt), but rather are found near neighboring Cantabria. Our results instead lend support to the hypothesis that the Basques are a distinct European population, with no detectable prehistoric connection to (or recent gene flow from) populations in the Caucasus or North Africa.
