# Supplementary material for: Autosomal short tandem repeat genetic variation of the Basques in Spain
Source: Croat Med J. 2011 Jun;52(3):372–83. doi: 10.3325/cmj.2011.52.372 (PMC3118713; doi:10.3325/cmj.2011.52.372)
Supplement: Supplementary Table 1 [file CroatMedJ_52_s003.pdf]

Supplementary Online Material. Basque Autosomal short tandem repeat frequencies by province.

Locus	Alava	Vizcaya	Guipuzcoa	Navarre
D3S1358	N = 96	N = 89	N = 154	N = 38
11	0.0052	0.0000	0.0000	0.0000
12	0.0052	0.0056	0.0097	0.0132
13	0.0208	0.0056	0.0065	0.0000
14	0.0833	0.1067	0.1429	0.0921
15	0.2969	0.2921	0.3442	0.2632
15.2	0.0052	0.0000	0.0000	0.0000
16	0.1875	0.3146	0.1786	0.2632
17	0.1771	0.1348	0.1071	0.1447
18	0.1927	0.1348	0.2045	0.2105
19	0.0260	0.0056	0.0065	0.0132
FGA				
17	0.0000	0.0112	0.0000	0.0000
18	0.0208	0.0449	0.0617	0.0395
19	0.0729	0.1292	0.1201	0.0921
20	0.1510	0.1461	0.1299	0.0658
20.2	0.0000	0.0000	0.0000	0.0132
21	0.2188	0.1292	0.1558	0.1842

21.2	0.0052	0.0000	0.0000	0.0000
22	0.1667	0.1348	0.0844	0.1447
22.2	0.000	0.0056	0.0032	0.0000
23	0.1094	0.1517	0.1916	0.1711
23.2	0.0052	0.0000	0.0000	0.0000
24	0.1458	0.1180	0.1234	0.1447
25	0.0781	0.0674	0.0942	0.1053
26	0.0208	0.0506	0.0195	0.0395
27	0.0000	0.0000	0.0065	0.0000
28	0.0052	0.0112	0.0065	0.0000
29	0.0000	0.0000	0.0032	0.0000
D5S818				
8	0.0052	0.0056	0.0032	0.0000
9	0.0208	0.0393	0.0422	0.0132
10	0.0885	0.0955	0.1104	0.0658
11	0.3854	0.3876	0.3442	0.2895
12	0.3490	0.2978	0.3052	0.4079
13	0.1406	0.1629	0.1883	0.2237
14	0.0052	0.0112	0.0065	0.0000
15	0.0052	0.0000	0.0000	0.0000

D7S820				
7	0.0521	0.0169	0.0130	0.0526
8	0.0938	0.1966	0.1623	0.1974
9	0.1198	0.1236	0.1039	0.0789
10	0.3385	0.2191	0.2857	0.2500
11	0.1823	0.2640	0.2500	0.1711
12	0.1354	0.1180	0.1364	0.2105
13	0.0521	0.0562	0.0292	0.0263
14	0.0260	0.0056	0.0195	0.0132
D8S1179				
8	0.0208	0.0169	0.0162	0.0000
9	0.0260	0.0169	0.0195	0.0000
10	0.0833	0.0899	0.0747	0.1053
11	0.0469	0.0281	0.0422	0.0658
12	0.0885	0.0787	0.1234	0.1184
13	0.2656	0.2809	0.3052	0.3026
14	0.3333	0.3258	0.2500	0.2105
15	0.1198	0.1517	0.1494	0.1711
16	0.0104	0.0056	0.0195	0.0263

17	0.0052	0.0056	0.0000	0.0000
vWA				
12	0.0000	0.0000	0.0000	0.0132
13	0.0000	0.0000	0.0000	0.0132
14	0.1458	0.1517	0.1136	0.0921
15	0.1563	0.1180	0.1331	0.1974
16	0.2344	0.2472	0.1883	0.1974
17	0.2708	0.2416	0.3344	0.2632
18	0.1458	0.1629	0.1331	0.1579
19	0.0469	0.0730	0.0909	0.0658
20	0.0000	0.0000	0.0065	0.0000
21	0.0000	0.0056	0.0000	0.0000
D13S317				
8	0.1875	0.1910	0.2175	0.1842
9	0.0365	0.0449	0.0455	0.0000
10	0.0365	0.0787	0.0227	0.0395
11	0.2604	0.2753	0.3247	0.3816
12	0.3646	0.2921	0.2500	0.2500
13	0.0781	0.0674	0.1039	0.1053

14	0.0365	0.0449	0.0325	0.0263
15	0.0000	0.0056	0.0032	0.0132
D18S51				
10	0.0208	0.0225	0.0130	0.0132
11	0.0156	0.0112	0.0227	0.0132
12	0.2188	0.1742	0.1786	0.1053
13	0.0990	0.0899	0.1558	0.1053
14	0.1198	0.1742	0.1396	0.1579
15	0.1406	0.1742	0.1623	0.1053
15.2	0.0000	0.0000	0.0032	0.0000
16	0.1146	0.1292	0.1136	0.1711
17	0.1615	0.0899	0.0877	0.2105
18	0.0469	0.0225	0.0422	0.0132
19	0.0313	0.0506	0.0130	0.0921
20	0.0156	0.0337	0.0390	0.0132
21	0.0052	0.0112	0.0195	0.0000
22	0.0052	0.0056	0.0097	0.0000
24	0.0052	0.0112	0.0000	0.0000
D21SS11				

26	0.0000	0.0056	0.0032	0.0000
27	0.0260	0.0393	0.0162	0.0395
28	0.1042	0.0787	0.0812	0.0921
29	0.1719	0.1685	0.2045	0.1579
29.2	0.0000	0.0056	0.0000	0.0000
30	0.2760	0.3146	0.2727	0.3289
30.2	0.0208	0.0843	0.0357	0.0263
31	0.0469	0.0506	0.0779	0.0132
31.2	0.0885	0.0843	0.0779	0.1316
32.0	0.0000	0.0000	0.0097	0.0000
32.2	0.1719	0.0843	0.1429	0.1711
33.2	0.0833	0.0730	0.0747	0.0395
34.2	0.0104	0.0056	0.0000	0.0000
35.2	0.0000	0.0056	0.0032	0.0000
